# Intensive Care Unit Occupancy in Japan, 2015–2018: A Nationwide Inpatient Database Study

**DOI:** 10.2188/jea.JE20210016

**Published:** 2022-12-05

**Authors:** Hiroyuki Ohbe, Yusuke Sasabuchi, Ryosuke Kumazawa, Hiroki Matsui, Hideo Yasunaga

**Affiliations:** 1 Department of Clinical Epidemiology and Health Economics, School of Public Health, The University of Tokyo, Tokyo, Japan; 2 Data Science Center, Jichi Medical University, Tochigi, Japan

**Keywords:** occupancy, intensive care unit, Japan, mechanical ventilation, extracorporeal membrane oxygenation

## Abstract

**Background:**

Detailed data on intensive care unit (ICU) occupancy in Japan are lacking. Using a nationwide inpatient database in Japan, we aimed to assess ICU bed occupancy to guide critical care utilization planning.

**Methods:**

We identified all ICU patients admitted from January 1, 2015 to December 31, 2018 to ICU-equipped hospitals participating in the Japanese Diagnosis Procedure Combination inpatient database. We assessed the trends in daily occupancy by counting the total number of occupied ICU beds on a given day divided by the total number of licensed ICU beds in the participating hospitals. We also assessed ICU occupancy for patients with mechanical ventilation, patients with extracorporeal membrane oxygenation, and patients without life-supportive therapies.

**Results:**

Over the 4 study years, 1,379,618 ICU patients were admitted to 495 hospitals equipped with 5,341 ICU beds, accounting for 75% of all ICU beds in Japan. Mean ICU occupancy on any given day was 60%, with a range of 45.0% to 72.5%. Mean ICU occupancy did not change over the 4 years. Mean ICU occupancy was about 9% higher on weekdays than on weekends and about 5% higher in the coldest season than in the warmest season. For patients with mechanical ventilation, patients with extracorporeal membrane oxygenation, and patients without life-supportive therapies, mean ICU occupancy was 24%, 0.5%, and 30%, respectively.

**Conclusion:**

Only one-fourth of ICU beds were occupied by mechanically ventilated patients, suggesting that the critical care system in Japan has substantial surge capacity under normal temporal variation to care for critically ill patients.

## INTRODUCTION

Intensive care unit (ICU) occupancy affects care decisions for patients under routine circumstances,^1^^–^^5^ and is also associated with planning in disaster or pandemic situations.^6^^,^^7^ Currently, Japan has about five ICU beds per 100,000 population, which is fewer than most other developed countries^8^^–^^12^; thus, most clinicians and policymakers in Japan consider the country’s supply of beds for critically ill patients to be insufficient to meet demand—both under the normal conditions of temporal variation and in a disaster situation.^13^

However, there have been limited data to guide planning for critical care utilization in Japan. Data on ICU occupancy and on the occupancy of high care units (HCUs)—a potential alternative to ICUs—are lacking. The proportions of patients admitted to the ICU who require the important resources of mechanical ventilation (MV) or extracorporeal membrane oxygenation (ECMO) are also unknown.^14^^–^^16^ Furthermore, no previous work has clarified what proportion of patients admitted to the hospital need ICU beds immediately (eg, patients receiving life-supportive therapies) and what proportion of these patients could be treated outside the ICU (eg, patients without life-supportive therapies).^16^^–^^18^

All these details are essential for understanding the flexibility of the national ICU system, informing decisions on how to plan for normal variation in critical care demand, and guiding national disaster planning. Therefore, we aimed to assess ICU and HCU occupancy in Japan using a nationwide inpatient database.

## METHODS

### Data source

This study was a retrospective cohort study that used administrative data that are routinely collected in Japan. The Institutional Review Board of The University of Tokyo approved this study (approval number, 3501-3; December 25, 2017). No information allowing the identification of individual patients, hospitals, or physicians was obtained, and the requirement for informed consent was waived because of the anonymous nature of the data. We used the Japanese Diagnosis Procedure Combination inpatient database, which contains discharge abstracts and administrative claims data from more than 1,200 acute-care hospitals in Japan that voluntarily contribute to the database.^19^ The database includes the following patient-level data for all hospitalizations: age, sex, level of consciousness at admission, admission type, diagnoses recorded using *International Classification of Diseases, Tenth Revision* codes, daily procedures recorded using Japanese medical procedure codes, daily drug administrations, and discharge status. A previous study that examined the validity of the recorded procedures showed that the sensitivity and specificity of procedures exceeded 90%.^20^

We also used data from the facility information and statistics presented in the 2017 Survey of Medical Institutions.^21^ We combined this information with the data in the Japanese Diagnosis Procedure Combination inpatient database using a specific hospital identifier. The Survey of Medical Institutions included the hospital zip code, type of ward (eg, general, ICU, or HCU), number of hospital beds in each ward, whether the institution was an academic hospital, and whether the institution was a tertiary emergency hospital.

### Study population

We included all patients admitted from January 1, 2015 to December 31, 2018 to hospitals participating in the Japanese Diagnosis Procedure Combination inpatient database that had an ICU and/or an HCU and whose data were successfully combined with the 2017 Survey of Medical Institutions. In this study, an ICU was defined as a separate unit providing critical care services with at least one physician on site 24 hours per day, around-the-clock nursing, the equipment necessary to care for critically ill patients, and a nurse-to-patient ratio of >1:2.^22^ The definition of an HCU in this study was almost the same as an ICU, except the required nurse-to-patient ratio was 1:4 or 1:5. We did not include patients admitted to the pediatric ICU, neonatal ICU, or obstetric ICU in this study.

Admission type was categorized as elective surgery, emergency surgery, or nonsurgical/acute medical problem. We defined patients who were admitted to the ICU/HCU on the day of elective or emergency surgery with general anesthesia as elective or emergency surgery patients. On the basis of the ICU admission prioritization framework in the 2016 ICU admission, discharge, and triage guidelines,^16^ we defined patients without life-supportive therapies (eg, intracranial pressure monitoring, invasive mechanical ventilation, noradrenaline, extracorporeal membrane oxygenation, intra-aortic balloon pump, left ventricular assist device, or continuous renal replacement therapy) as having lower priority for ICU admission.

### Analysis of occupancy

We calculated the daily occupancy for all patients in the ICU/HCU, patients with MV, and patients with ECMO. We defined occupancy as the percentage of the total number of licensed ICU/HCU beds as reported in the 2017 Survey of Medical Institutions that were in use for the patient group being considered.

We assessed occupancy in two different ways. First, we calculated occupancy as the total number of beds occupied by patients in the cohort on a given day divided by the total number of licensed ICU/HCU beds in the participating hospitals in this study. We used this approach to assess temporal trends in overall occupancy during the study period. In this article, we present the findings from this part of the analysis graphically using plots of all daily data accompanied by the moving weekly averages over the study period. Using calendar year, we estimated linear regression models to assess whether occupancy increased over time. We also assessed differences in occupancy by day of the week (weekday versus weekend) and by the four seasons (January to March, April to June, July to September, and October to December). In addition, we extrapolated national estimates of the daily number of occupied beds by obtaining the number of ICU/HCU beds used by patients in the participating hospitals as a proportion of all ICU/HCU beds reported in the 2017 Survey of Medical Institutions. We also graphically presented the daily occupancy for patients without life-supportive therapies using plots of all daily data accompanied by the moving weekly averages over the study period.

Second, we calculated the mean daily ICU or HCU occupancy in each hospital as the mean total number of beds occupied by patients on a given day divided by the total number of licensed ICU/HCU beds in each hospital throughout the entire study period, and we summarized these data by presenting means and standard deviations (SDs) across all hospitals. We used this approach to assess the variation in occupancy between hospitals. We also assessed the variation in occupancy between academic and non-academic hospitals, between tertiary and non-tertiary emergency hospitals, and between hospitals with low, medium, and high bed volume.

### Statistical analysis

Continuous variables are presented as means and SDs or as medians and interquartile ranges (IQRs). Categorical variables are presented as numbers and percentages. All analyses were performed using Stata/MP, Version 16.0 (StataCorp, College Station, TX, USA).

## RESULTS

In the 2017 Survey of Medical Institutions, there were 629 ICU-equipped hospitals with 7,109 ICU beds and 679 HCU-equipped hospitals with 10,268 HCU beds in Japan, representing an estimated 5.6 ICU beds per 100,000 persons and 8.1 HCU beds per 100,000 persons in the country. After excluding the patients whose data were not combined with the 2017 Survey of Medical Institutions, the study cohort consisted of 2,740,559 patients admitted to 743 hospitals with 12,476 ICU/HCU beds (1,379,618 ICU patients admitted to 495 hospitals with 5,341 ICU beds and 1,536,800 HCU patients admitted to 513 hospitals with 7,161 HCU beds) over 4 years. A total of 75% (5,341/7,109 beds) of all ICU beds in Japan and 70% (7,161/10,268 beds) of all HCU beds in Japan were included in the present study. In the participating hospitals, there was a median of 8 total ICU beds (IQR: 6–14 beds) and a median of 12 total HCU beds (IQR: 8–22 beds).

The mean age was 66.5 (SD, 18.7) years for ICU patients and 69.8 (SD, 17.6) years for HCU patients (Table 1). About 60% of the patients were male. The percentages of patients who were admitted for elective surgery, emergency surgery, and nonsurgical/acute medical problems were 39%, 13%, and 47%, respectively, in the ICU and 18%, 9%, and 73%, respectively, in the HCU. Among the ICU patients admitted for elective surgery, 70% did not receive life-supportive therapies. The percentages of patients receiving MV and ECMO during an ICU/HCU stay were 32% and 1.1%, respectively, in the ICU and 14% and 0.2%, respectively, in the HCU. In-hospital mortality was 12% and the median length of ICU/HCU stay was 2 days (IQR, 1–4) in both the ICU and the HCU.

**Table 1.** Characteristics and outcomes of patients admitted to the ICU/HCU

Characteristic or outcome	ICU	HCU	ICU/HCU
	(*n* = 1,379,618)	(*n* = 1,536,800)	(*n* = 2,740,559)
Age, years, mean (SD)	66.5 (18.7)	69.9 (17.6)	68.3 (18.3)
Male, *n* (%)	834,457 (60)	876,566 (57)	1,602,204 (58)
Charlson comorbidity index, mean (SD)	1.2 (1.5)	1.1 (1.5)	1.1 (1.5)
Japan Coma Scale at admission, *n* (%)			
Alert	1,024,291 (74)	931,405 (61)	1,853,765 (68)
Dizziness	150,613 (11)	327,017 (21)	443,281 (16)
Somnolence	60,870 (4)	112,024 (7)	157,488 (6)
Coma	143,844 (10)	166,354 (11)	286,025 (10)
Admission type, *n* (%)			
Elective surgery	544,197 (39)	279,485 (18)	790,778 (29)
Emergency surgery	183,611 (13)	137,966 (9)	295,433 (11)
Nonsurgical/acute medical problem	651,810 (47)	1,119,349 (73)	1,654,348 (60)
Main etiologies for ICU/HCU admission, *n* (%)			
Circulatory diseases	648,946 (47)	601,660 (39)	1,161,804 (42)
Neoplasms and diseases of the blood	358,013 (26)	241,142 (16)	576,980 (21)
Injury, poisoning, or external causes	108,663 (8)	225,414 (15)	306,441 (11)
Abdominal diseases	107,892 (8)	171,995 (11)	262,954 (10)
Respiratory diseases	84,766 (6)	167,533 (11)	235,862 (9)
Treatments during ICU/HCU stay, *n* (%)			
Mechanical ventilation	414,003 (32)	196,186 (14)	633,054 (23)
Noradrenaline	329,746 (26)	103,138 (7)	455,257 (17)
Continuous renal replacement therapy	57,977 (5)	12,727 (0.9)	74,044 (3)
Intra-aortic balloon pumping	37,440 (3)	9,058 (0.6)	48,735 (2)
Extracorporeal membrane oxygenation	14,714 (1.1)	2,755 (0.2)	18,187 (0.7)
Intracranial pressure monitoring	3,994 (0.3)	1,058 (0.1)	5,488 (0.2)
Left ventricular assist device	230 (0.0)	20 (0.0)	261 (0.0)
No life-supportive therapies	731,470 (57)	1,190,133 (82)	1,892,699 (69)
Discharge status, *n* (%)			
In-hospital death	167,875 (12)	188,316 (12)	338,453 (12)
Discharge to home	941,202 (68)	951,875 (62)	1,798,406 (66)
Transfer to another hospital	247,194 (18)	328,269 (21)	515,917 (19)
Transfer to a nursing facility	22,060 (2)	65,622 (4)	83,979 (3)
Missing	1,287 (0.1)	2,718 (0.2)	3,804 (0.1)
Length of ICU/HCU stay, days, mean (SD)	2 (1, 4)	2 (1, 4)	2 (1, 4)
Length of stay, days, median (IQR)	19 (11, 33)	15 (8, 28)	16 (9, 30)
Hospitalization costs, 10,000 yen, mean (SD)	200 (126, 348)	121 (65, 201)	152 (82, 257)

HCU, high care unit; ICU, intensive care unit; IQR, interquartile range; SD, standard deviation.

**Figure 1.**
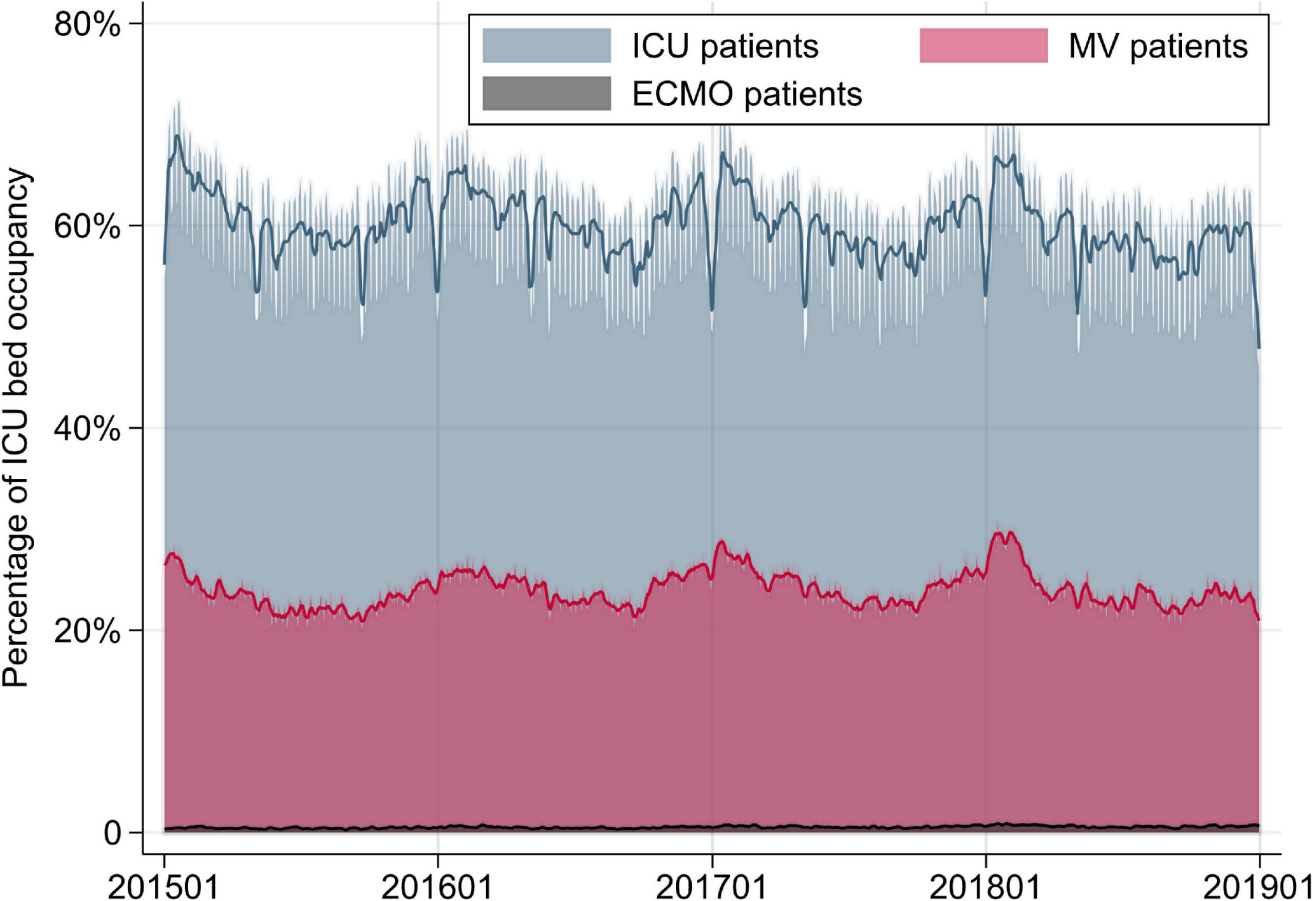
Percentages of ICU bed occupancy for all patients in the ICU, patients receiving MV, and patients receiving ECMO from January 1, 2015 to December 31, 2018. Smoothed lines show 1-week moving averages. ECMO, extracorporeal membrane oxygenation; ICU, intensive care unit; MV, mechanical ventilation.

**Figure 2.**
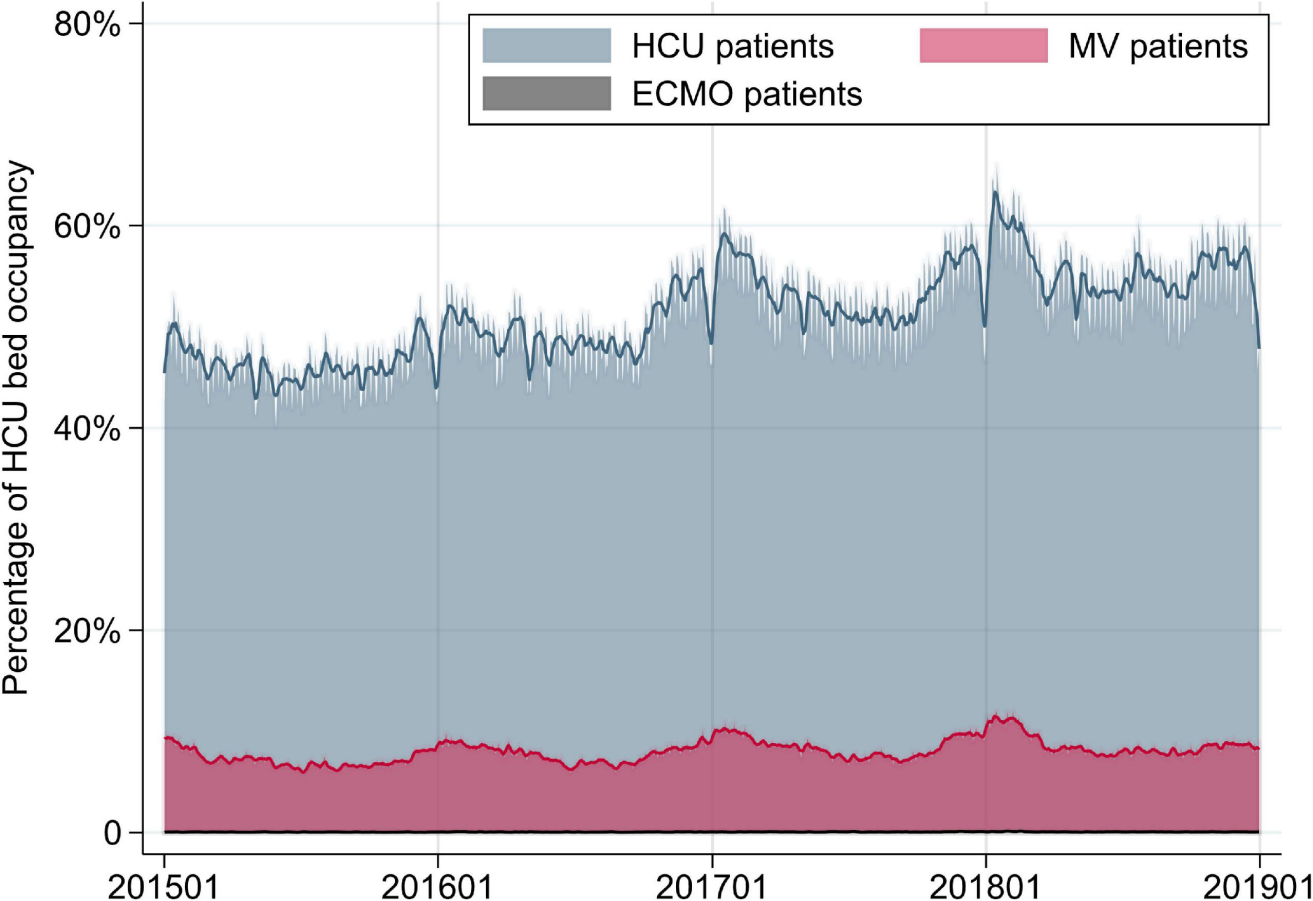
Percentages of HCU bed occupancy for all patients in the HCU, patients receiving MV, and patients receiving ECMO from January 1, 2015 to December 31, 2018. Smoothed lines show 1-week moving averages. ECMO, extracorporeal membrane oxygenation; HCU, high care unit; MV, mechanical ventilation.

Mean ICU occupancy for all ICU patients, MV patients, and ECMO patients was 60.0%, 24.0%, and 0.53%, respectively (Table 2 and Figure 1). Mean HCU occupancy for all HCU patients, MV patients, and ECMO patients on any given day was 51.3%, 8.0%, and 0.05%, respectively (Table 2 and Figure 2). The overall mean ICU occupancy did not change over the 4 study years, whereas mean ICU occupancy for MV patients increased over this time period. Mean HCU occupancy also increased over the 4 study years. Mean ICU occupancy was about 9% higher on weekdays than on weekends. Mean ICU occupancy was higher in the coldest season than in the warmest season both for all ICU patients (about 5% higher) and for MV patients (about 4% higher). Extrapolating our findings to national estimates of the daily numbers of patients in Japan, there were a total of 3,926 patients in the ICU, including 1,578 MV patients and 35 ECMO patients, and there were 4,763 patients in the HCU, including 743 MV patients and 5 ECMO patients.

**Table 2.** Temporal trends in ICU and HCU occupancy

	ICU/HCU patients	Difference (95% CI)	MV patients	Difference (95% CI)	ECMO patients	Difference (95% CI)
**ICU**						

Overall occupancy, %, mean (SD)	60.0 (5.6)		24.0 (1.9)		0.53 (0.14)	
Overall occupancy, %, min, max	45.0, 72.5		20.0, 30.7		0.17, 1.09	
Year, %, mean (SD)						
2015	60.6 (5.5)	Base	23.2 (1.7)	Base	0.44 (0.10)	Base
2016	60.6 (5.4)	−0.1 (−0.9, 0.7)	24.3 (1.5)	1.1 (0.8, 1.3)	0.50 (0.11)	0.06 (0.04, 0.08)
2017	59.8 (5.5)	−0.8 (−1.6, 0.0)	24.5 (1.8)	1.3 (1.0, 1.5)	0.56 (0.12)	0.12 (0.11, 0.14)
2018	59.1 (5.7)	−1.5 (−2.3, −0.7)	24.1 (2.2)	0.9 (0.6, 1.1)	0.63 (0.14)	0.18 (0.17, 0.20)
Day of the week, %, mean (SD)						
Weekday (Monday to Friday)	62.6 (4.1)	Base	24.2 (1.9)	Base	0.55 (0.14)	Base
Weekend (Saturday or Sunday)	53.6 (3.0)	−9.0 (−9.4, −8.5)	23.6 (1.9)	−0.6 (−0.8, −0.4)	0.49 (0.13)	−0.06 (−0.07, −0.04)
Season, %, mean (SD)						
January to March	63.0 (5.3)	5.4 (4.6, 6.1)	25.9 (1.8)	3.5 (3.3, 3.7)	0.60 (0.16)	0.13 (0.11, 0.15)
April to June	59.4 (5.3)	1.7 (1.0, 2.5)	23.5 (1.3)	1.0 (0.8, 1.2)	0.50 (0.12)	0.03 (0.01, 0.05)
July to September	57.6 (4.9)	Base	22.5 (1.0)	Base	0.47 (0.12)	Base
October to December	60.1 (5.4)	2.5 (1.7, 3.3)	24.2 (1.4)	1.7 (1.5, 1.9)	0.56 (0.13)	0.09 (0.08, 0.11)

**HCU**						

Overall occupancy, %, mean (SD)	51.3 (4.8)		8.0 (1.1)		0.05 (0.03)	
Overall occupancy, %, min, max	40.1, 66.1		5.7, 12.1		0.00, 0.18	
Year, %, mean (SD)						
2015	46.2 (2.4)	Base	7.2 (0.8)	Base	0.04 (0.02)	Base
2016	49.7 (3.1)	3.5 (3.0, 4.0)	7.8 (0.9)	0.6 (0.5, 0.7)	0.05 (0.03)	0.01 (0.00, 0.01)
2017	53.5 (3.6)	7.2 (6.7, 7.7)	8.5 (1.0)	1.3 (1.1, 1.4)	0.06 (0.03)	0.02 (0.02, 0.02)
2018	55.6 (3.7)	9.4 (8.9, 9.8)	8.7 (1.1)	1.5 (1.3, 1.6)	0.06 (0.03)	0.02 (0.02, 0.03)
Day of the week, %, mean (SD)						
Weekday (Monday to Friday)	52.4 (4.7)	Base	8.1 (1.1)	Base	0.05 (0.03)	Base
Weekend (Saturday or Sunday)	48.5 (3.9)	−3.9 (−4.4, −3.4)	7.9 (1.1)	−0.1 (−0.3, 0.0)	0.05 (0.03)	−0.01 (−0.01, 0.00)
Season, %, mean (SD)						
January to March	52.6 (5.4)	3.0 (2.3, 3.7)	9.0 (1.2)	1.9 (1.7, 2.0)	0.06 (0.03)	0.01 (0.01, 0.02)
April to June	50.0 (4.2)	0.3 (−0.3, 1.0)	7.7 (0.7)	0.5 (0.3, 0.6)	0.05 (0.03)	0.00 (0.00, 0.00)
July to September	49.6 (4.0)	Base	7.2 (0.7)	Base	0.05 (0.03)	Base
October to December	52.8 (4.8)	3.1 (2.5, 3.8)	8.3 (0.9)	1.1 (1.0, 1.2)	0.05 (0.03)	0.00 (0.00, 0.01)

CI, confidence interval; ECMO, extracorporeal membrane oxygenation; HCU, high care unit; ICU, intensive care unit; MV, mechanical ventilation; SD, standard deviation.

Occupancies were calculated as the total number of beds occupied by patients in the cohort on a given day divided by the total number of licensed ICU/HCU beds in the participating hospitals in this study.

**Figure 3.**
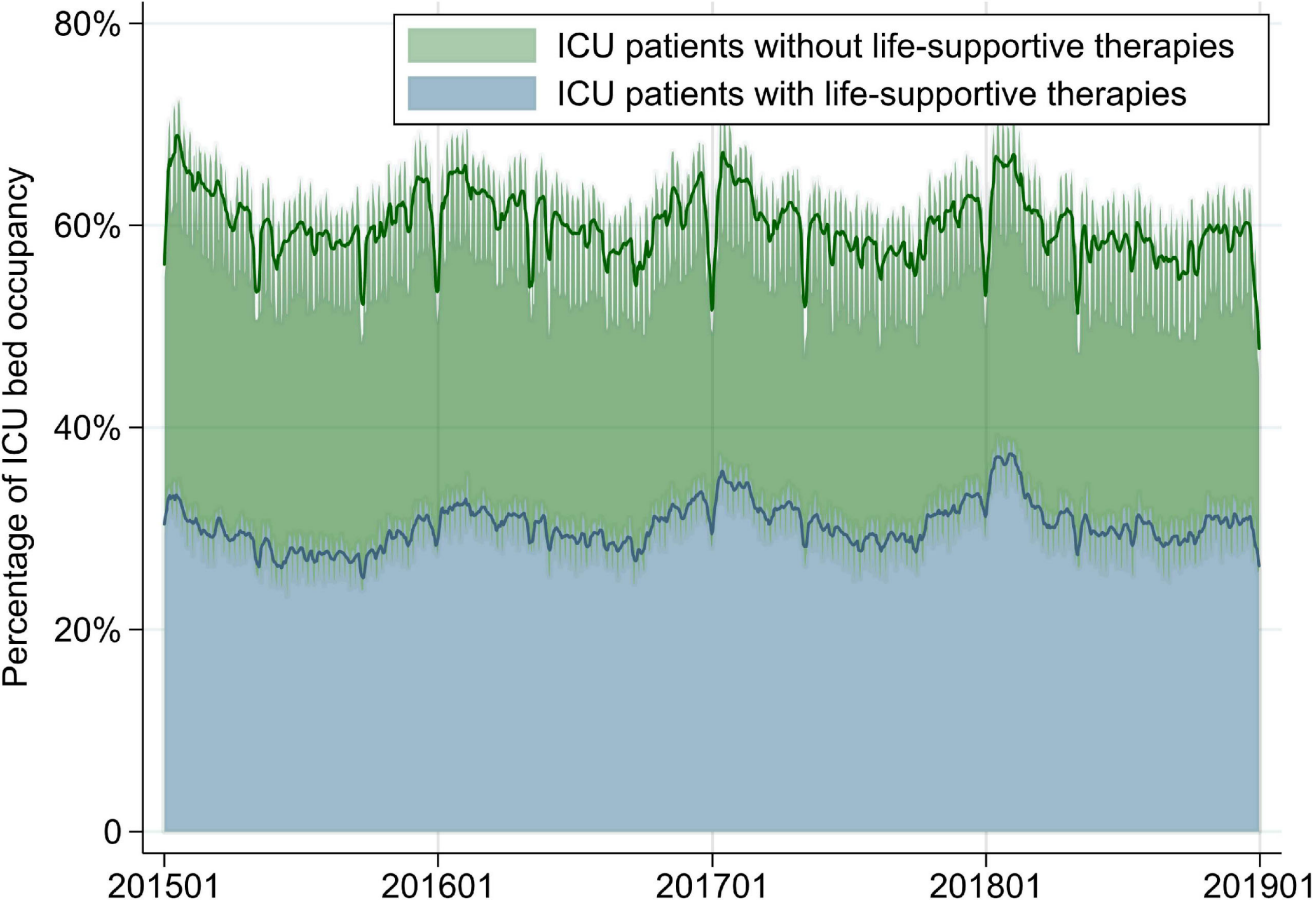
Percentages of ICU bed occupancy for all patients in the ICU and patients without life-supportive therapies from January 1, 2015 to December 31, 2018. Smoothed lines show 1-week moving averages. ICU, intensive care unit.

**Figure 4.**
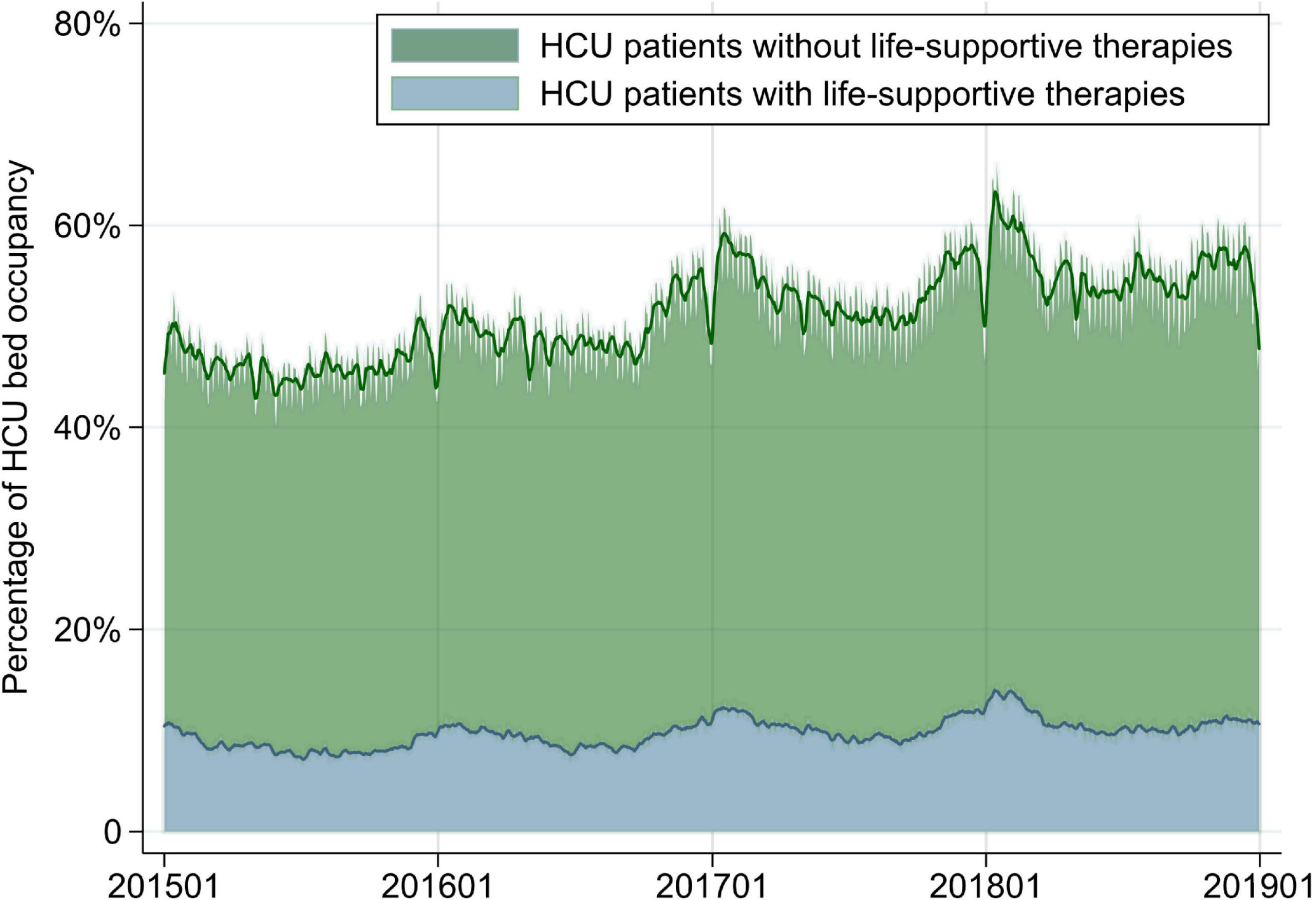
Percentages of HCU bed occupancy for all patients in the HCU and patients without life-supportive therapies from January 1, 2015 to December 31, 2018. Smoothed lines show 1-week moving averages. HCU, intensive care unit.

Mean occupancy for patients without life-supportive therapies was 29.7% (SD, 3.7%) in ICUs and 41.5% (SD, 3.7%) in HCUs (Figure 3 and Figure 4). Mean ICU occupancy varied widely across hospitals for ICU patients (range, 21.1–112.0%), MV patients in the ICU (range, 0.0–62.7%), and ECMO patients in the ICU (range, 0.0–3.0%) (Table 3). There were no differences in mean ICU occupancy between academic and non-academic hospitals or between tertiary and non-tertiary emergency hospitals, whereas mean ICU occupancy for MV patients in the ICU was approximately 3% higher in academic hospitals than in non-academic hospitals and approximately 5% higher in tertiary emergency hospitals than in non-tertiary emergency hospitals. Lower ICU bed volume was associated with higher overall ICU occupancy but not with higher occupancy for MV patients in the ICU.

**Table 3.** Variation in mean daily ICU and HCU occupancy between hospitals

	Number of hospitals	ICU/HCU patients	Difference (95% CI)	MV patients	Difference (95% CI)	ECMO patients	Difference (95% CI)
**ICU**							

Mean daily occupancy, %, mean (SD)	495	58.6 (14.4)		22.7 (9.1)		0.46 (0.51)	
Mean daily occupancy, %, min, max	495	21.1, 112.0		0.0, 62.7		0.0, 3.0	
Type of hospital, %, mean (SD)							
Non-academic	416	59.1 (14.8)	Base	22.3 (9.1)	Base	0.41 (0.47)	Base
Academic	79	56.4 (12.3)	−2.7 (−6.1, 0.8)	25.1 (9.1)	2.8 (0.6, 5.0)	0.73 (0.62)	0.32 (0.20, 0.44)
Type of emergency hospital, %, mean (SD)							
Non-tertiary	275	58.7 (14.3)	Base	20.6 (8.9)	Base	0.30 (0.38)	Base
Tertiary	220	58.6 (14.7)	−0.2 (−2.7, 2.4)	25.4 (8.7)	4.8 (3.2, 6.3)	0.67 (0.57)	0.37 (0.28, 0.45)
ICU bed volume, %, mean (SD)							
Low (2–7 beds)	149	63.8 (14.2)	Base	22.9 (10.3)	Base	0.32 (0.41)	Base
Medium (8–11 beds)	172	57.6 (14.6)	−6.3 (−9.4, −3.2)	22.4 (9.3)	−0.5 (−2.5, 1.5)	0.48 (0.58)	0.15 (0.04, 0.26)
High (12–70 beds)	174	55.3 (13.3)	−8.5 (−11.6, −5.4)	22.9 (7.8)	0.0 (−2.0, 2.0)	0.56 (0.49)	0.24 (0.13, 0.35)

**HCU**							

Mean daily occupancy, %, mean (SD)	513	53.8 (24.2)		9.3 (6.1)		0.06 (0.17)	
Mean daily occupancy, %, min, max	513	7.7, 126.9		0.0, 34.2		0.0, 2.1	
Type of hospital, %, mean (SD)							
Non-academic	457	54.5 (23.0)	Base	9.5 (6.1)	Base	0.06 (0.17)	Base
Academic	56	48.6 (32.5)	−5.8 (−12.5, 0.9)	7.2 (5.8)	−2.3 (−4.0, −0.6)	0.08 (0.16)	0.02 (−0.03, 0.06)
Type of emergency hospital, %, mean (SD)							
Non-tertiary	289	60.0 (24.1)	Base	10.8 (6.5)	Base	0.06 (0.19)	Base
Tertiary	224	46.0 (22.0)	−14.0 (−18.1, −9.9)	7.3 (5.0)	−3.6 (−4.6, −2.6)	0.06 (0.14)	0.00 (−0.03, 0.03)
HCU bed volume, %, mean (SD)							
Low (4–7 beds)	113	68.7 (35.0)	Base	13.3 (7.5)	Base	0.10 (0.26)	Base
Medium (8–19 beds)	229	54.2 (17.6)	−14.4 (−19.6, −9.3)	9.4 (5.6)	−3.8 (−5.1, −2.6)	0.06 (0.16)	−0.04 (−0.08, 0.00)
High (20–78 beds)	171	43.6 (17.5)	−25.1 (−30.5, −20.0)	6.5 (3.9)	−6.8 (−8.1, −5.4)	0.05 (0.10)	−0.05 (−0.09, −0.01)

CI, confidence interval; ECMO, extracorporeal membrane oxygenation; HCU, high care unit; ICU, intensive care unit; MV, mechanical ventilation; SD, standard deviation.

Mean daily ICU or HCU occupancy in each hospital was calculated as the mean total number of beds occupied by patients on a given day divided by the total number of licensed ICU/HCU beds in each hospital throughout the entire study period, and these data are summarized as means and standard deviations across all hospitals.

## DISCUSSION

The present study provided detailed nationwide findings on ICU occupancy in Japan. Approximately 60% of ICU beds were occupied by all ICU patients, 25% were occupied by MV patients, and 30% were occupied by patients who did not receive life-supportive therapies.

ICU bed occupancy in Japan was lower than we anticipated. A previous study of 97 ICUs in the United States showed that approximately 70% of ICU beds were occupied and that approximately 30% of these beds were occupied by patients requiring MV.^14^ The results of the present study were surprising because compared with Japan, the United States has four times more ICU beds—approximately 20 per 100,000 population.^9^^,^^10^ Currently, there appears to be no definitive evidence on what the optimal target for ICU occupancy should be. Previous studies have shown higher ICU occupancy, particularly above 80%, to be associated with increased mortality and ICU readmission.^1^^–^^5^ Physicians’ consensus statements in the previous literature suggest that optimal ICU occupancy may be around 70–75% for ICU beds for critically ill patients.^23^^–^^25^ Therefore, our results may indicate that the health care system in Japan has substantial surge capacity to care for acutely critically ill patients under normal temporal variation.

There are several possible options for responding to surges in demand for care for critically ill patients during a disaster or pandemic: critical care triage and rationing of resources, provision of intensive care outside the ICU, rapidly building new hospitals with ICUs, and centralization of critically ill patients in designated hospitals with ICUs.^18^^,^^26^ Our results suggest that many patients who are not receiving life-supportive therapies in the ICU could be treated outside the ICU. Therefore, we can reduce strain on ICUs considerably by postponing elective surgeries and by treating lower-acuity patients in other areas of the hospital, such as HCUs, and these strategies should be considered preferred options. Another option to reduce strain on ICUs would be having HCU beds function as ICU beds. However, it may be better not to expect HCUs to fulfill too many of the functions usually carried out by ICUs because increasing the ICU-level workload without increasing the number of staff members might result in increased mortality.^27^ Thus, the conversion of HCUs to ICUs should be accompanied by an appropriate increase in staffing, shifting the nurse-to-patient ratio from 1:4 to 1:2. For the reasons mentioned above, increasing the total supply of ICU beds by building new hospitals with ICUs may not be a preferred option under the current situation of low ICU occupancy.

Life-saving equipment is also essential for critical care capacity.^18^^,^^28^^,^^29^ As of 2020, there were an estimated 45,000 ventilators and 2,200 ECMO devices in Japan.^30^ Assuming that MV and ECMO are exclusively performed in ICUs and/or HCUs, the estimated daily utilization rates for MV and ECMO in Japan are 5% (2,321/45,000) and 2% (45/2,200), respectively. Although life-saving equipment in Japan appears to be sufficient, there has been a shortage of nurses skilled in ICU care in the country. A previous report showed that in Japan, the nurse-to-patient ratio was lower than 1:2 in more than 80% of ICUs and that critical care nurses made up less than 10% of round-the-clock nurse staffing.^31^ Therefore, our data imply that appropriately trained staff members who can manage these life-supportive therapies are far more important, compared with the fixed resources of MV or ECMO, in the provision of critical care in Japan.

The present study had several strengths. First, this study successfully created a nationally representative cohort of a large number of ICU and HCU patients (representing 75% of all ICU beds and 70% of all HCU beds in Japan). To the best of our knowledge, this is the first study to assess ICU and HCU occupancy in Japan on a national scale. Furthermore, we were able to assess the use of MV and ECMO using well-validated procedure codes.^20^

However, the present study also had several limitations. First, the calculated occupancy rates depended on the numbers of ICU and HCU beds that were billed through insurance claims. However, the cost of ICU and HCU stays can be billed only for up to 14 days and 21 days, respectively. Thus, the calculated occupancy rates in our study may be underestimated. Second, to calculate occupancy, we used the total number of licensed ICU and HCU beds reported in the 2017 Survey of Medical Institutions, but some hospitals may have decreased or increased their number of ICU/HCU beds during the 4 study years, resulting in an underestimation or overestimation of occupancy.

In conclusion, using a nationwide inpatient database, we revealed that ICU occupancy in Japan did not increase over the 4 study years but that there was considerable variation from day to day and from season to season. Only one-fourth of ICU beds were occupied by MV patients, suggesting that Japan’s critical care system has substantial surge capacity to care for critically ill patients under normal temporal variation. However, whether this capacity can be exploited in a disaster situation requires further multidisciplinary studies. Our data imply that postponing elective surgeries and treating lower-acuity patients in other areas of the hospital, such as HCUs, are preferred options for reducing strain on ICUs when responding to a surge in demand for care for critically ill patients during a disaster or pandemic.
